# Protein kinase C β inhibits autophagy and sensitizes cervical cancer Hela cells to cisplatin

**DOI:** 10.1042/BSR20160445

**Published:** 2017-03-27

**Authors:** Na Li, Wei Zhang

**Affiliations:** Department of Gynaecology and Obstetrics, Zhongnan Hospital of Wuhan University, Wuhan 430071, China

**Keywords:** Autophagy, Chemotherapy, Cisplatin, Protein kinase C β

## Abstract

Recently, autophagy has been indicated to play an essential role in various biological events, such as the response of cervical cancer cells to chemotherapy. However, the exact signalling mechanism that regulates autophagy during chemotherapy remains unclear. In the present study, we investigated the regulation by cisplatin on protein kinase C β (PKC β), on B-cell lymphoma 2 (Bcl-2) and on apoptosis in cervical cancer Hela cells. And then we examined the regulation by cisplatin on autophagy and the role of autophagy on the chemotherapy in Hela cells. In addition, the regulation of the PKC β on the autophagy was also investigated. Our results indicated that cisplatin promoted PKC β in Hela cells. The PKC β inhibitor reduced the cisplatin-induced apoptosis, whereas increased the cisplatin-induced autophagy in Hela cells. On the other side, the PKC β overexpression aggravated the cisplatin-induced apoptosis, whereas down-regulated the cisplatin-induced autophagy. Taken together, our study firstly recognized the involvement of PKC β in the cytotoxicity of cisplatin via inhibiting autophagy in cervical cancer cells. We propose that PKC β would sensitize cervical cancer cells to chemotherapy via reducing the chemotherapy induced autophagy in cancer cells.

## Introduction

Cisplatin is an effective chemotherapeutic agent that is widely applied in treating solid tumours such as bladder, head and neck, lung, ovarian and testicular cancers [1]. Besides its side effects, acquired resistance to the cisplatin of tumour emerges during the course of treatment limits its application [2–4], however, cisplatin combination chemotherapy is still the basis of chemotherapy against many cancers. To our knowledge, the proposed mechanisms of chemotherapy resistance include changes in cellular uptake and release of agent, improved biotransformation, antiapoptotic mechanisms and autophagy [5–8].

Autophagy is a type of non-apoptotic cell death that degrades unnecessary or dysfunctional cellular components through the actions of lysosomes, and is essential for survival when cells faced a harsh environment such as radiation and chemotherapy [9]. During the autophagy, targeted cytoplasmic constituents are packed into a double-membraned vesicle (autophagosome), which is fused with lysosomes to generate autolysosomes and is degraded consequently [10]. It was found that chemotherapy activates autophagy in tumour cells, which has been considered to enhance cancer cell death or play a role in chemotherapy resistance [11,12].

The protein kinase C (PKC) family consists of at least 12 kinases with distinct roles in cell proliferation, differentiation and apoptosis [13]. PCK family plays a key role in tumour promotion and progression and it is an ideal target for cancer therapy [14].PCK β activation blocks insulin-induced endothelial nitric oxide synthase (eNOS) stimulation, whereas inhibition of PKC β restores vascular function in animal models [15]. In addition, the improved PKC β activity has also been found implicating in activating endothelial cell inflammatory [16].

Cisplatin activates several signal transduction pathways mediated by ROS, DNA damage, p53, TNFα and MAPKs [17], yet the specific pathways involved in autophagy relating to cisplatin are unknown, the relationship between autophagy and apoptosis in cancer cells are still unclear and the signalling mechanism that regulates autophagy during chemotherapy is to be explored. In this research, we investigated the regulation by cisplatin on PKC β, on B-cell lymphoma 2 (Bcl-2) and on apoptosis in cervical cancer Hela cells, examined the regulation by cisplatin on autophagy and the role of autophagy on the chemotherapy and investigated the regulation of the PKC β on the autophagy.

## Materials and methods

### Cell culture

The cervical cancer Hela cell was purchased from ATCC. All cell lines were cultured in Dulbecco’s modified Eagle’s medium (DMEM) containing 10% FBS (Gibco, U.S.A.) in the humidified air with 5% CO_2_ at 37°C. Cisplatin was purchased from Sigma–Aldrich (St. Louis, U.S.A.) and was dissolved in DMEM with a storage concentration of 1 mM. Enzastaurin (Sigma–Aldrich, U.S.A.) was utilized to inhibit PKC β. Rapamycin (Rapa) (Thermo Fisher Scientific, U.S.A.) and 3-Methyladenine (3-MA) (Sigma-Aldrich, U.S.A.) were utilized as positive and negative autophagy activators respectively. To overexpress PCK β, the PCK β-pcDNA3.1(+) (Sino Biological, Beijing, China) was transfected with Lipofectamine 2000 (Invitrogen, Carlsbad, CA, U.S.A.) into Hela cells.

### Assessment of cellular viability using MTT assay

Hela cells (2 × 10^3^) in the log-growth phase were seeded in a 96-well plate. After overnight growth, the cells were treated with different doses of cisplatin/enzastaurin/PKC β. MTT (20 μl) in the final concentration of 5 mg/ml was added and the cells were incubated for 4 h at 37°C. After the supernatant was carefully removed, 200 μl of DMSO was added into each well and mixed. The plate was put into a 37°C incubator to dissolve air bubbles and OD_570_ value of each well was measured at 570 nm wavelength using a microplate reader (Thermo Scientific, U.S.A.). The results were expressed as (A_570_ of control wells – A_570_ of treated wells)/(A_570_ of control wells – A_570_ of blank wells) × 100%.

### Western blot analysis

Treated cells were lysed with lysis buffer (Invitrogen, U.S.A.) on ice for 20 min. The cell lysates were centrifugated at 13000 × ***g*** at 4°C for 30min, the supernatant was collected as the total cellular protein extract. Protein concentration was determined using the BCA Protein Assay Kit (Kangwei Shiji Company, Beijing). Samples of total cellular protein were loaded on to 10% SDS/PAGE. The separated proteins were electrophoretically transferred to PVDF membranes (Bio-Rad, U.S.A.). The membrane was blocked overnight in blocking buffer containing PBS-T and 5% non-fatty milk. Then the membrane was incubated with primary antibody against different antigens for 1 h separately and was washed with PBST for four times subsequently. Following incubating with the secondary HRP-conjugated antibody for 1 h, the PVDF membrane was washed for four times and was treated with ECL reagent and exposed to X-ray film. Each band was quantified using Image software. The protein level of each molecule was calculated according to its band intensity to glyceraldehyde-3-phosphate dehydrogenase (GAPDH) band intensity and was averaged for three independent experiments.

### Apoptosis assay

Cells apoptotic percentage was performed by AnnexinV-FITC Apoptosis Detection Kit (Merk Company, U.S.A.). Briefly, the treated cells were centrifugated at 1000 × ***g*** for 5 min and washed twice with PBS, then the cells were suspended in the 400 μl 1× binding buffer at a concentration of 1 × 10^5^ cells/ml, then 5 μl of Annexin V-FITC and propidium iodide was added in turn and mixed, the treated cells were placed in the dark at RT for 5–15 min to perform flow cytometry analysis.

### Detection of autophagic punctas by fluorescence microscopy

Detection of autophagic punctas was performed as previously described [18]. Briefly, treated Hela cells were seeded on sterile coverslips and incubated under the conditions mentioned above. The growth medium was removed and cells were washed with cold PBS for two times when the cells’ confluence reached 80%. Then, fresh growth medium was supplemented subsequently and cells were incubated for an additional 12 h. Detection of GFP–LC3 was performed using FlowCellect™ GFP–LC3 Reporter Autophagy Assay kit (Millipore, U.S.A.). Briefly, 10 μl autophagy reagent A was added and cells were incubated in a humidified incubator at 37°C with 5% CO_2_ for 2 h. Post the removal of medium, cells were washed with 5 ml 1× HBSS, were added with 100 ml 1× autophagy reagent B, and then were cultured with cells for 5 min, followed by washing with assay buffer for the autophagy reagent removal. Last, the coverslips were covered by sterile slides and the slides were observed under an Olympus fluorescence microscope (BX51, Olympus Corporation, Japan).

### Statistical analysis

Data are depicted as the mean ± S.D. For a comparison between the two groups, Student’s *t* test was used. For multiple comparisons among three or more groups, one-way ANOVA was used. *P*<0.05 was considered as a statistically significant difference.

## Results

### Cisplatin activates PKC β and down-regulates Bcl-2

**Figure 1:**
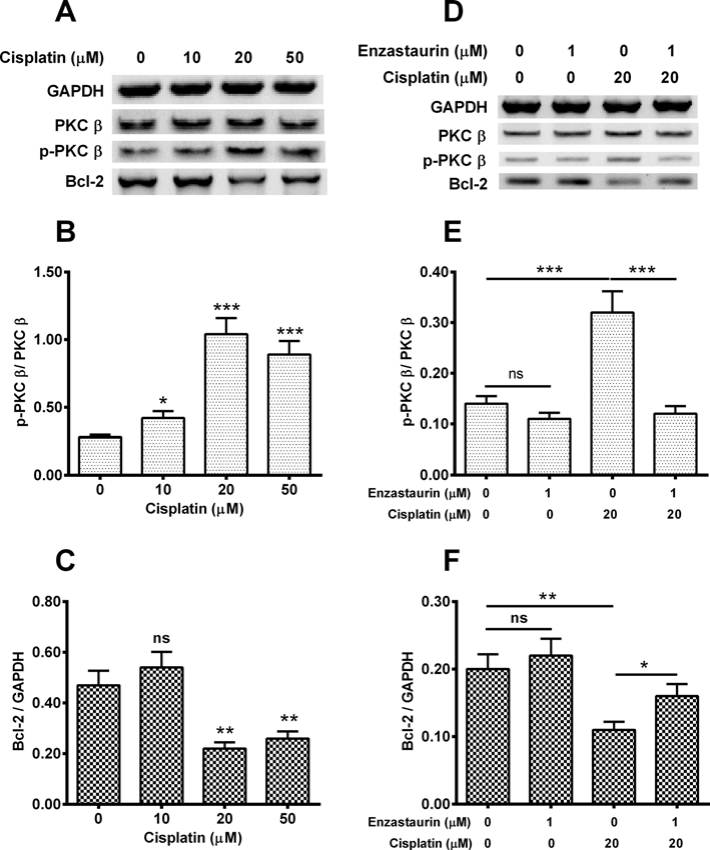
Cisplatin activates PKC β and down-regulates Bcl-2 in cervical cancer Hela cell line Eighty five percent confluent Hela cells were treated with 0, 10, 20 or 50 μM cisplatin, and with 0 or 1 μM PKC β inhibitor, enzastaurin for 24 h, then the expression of PKC β and Bcl-2, the phosphorylation of PKC β (T642). (**A**) Western blotting assay for PKC β, Bcl-2 and p-PKC β (with GAPDH as an internal control) in the cisplatin-treated Hela cells; (**B**, **C**): Ratio of p-PKC β to PKC β, of Bcl-2 to GAPDH in the cisplatin-treated Hela cells; (**D**) Western blotting assay for PKC β, Bcl-2 and p-PKC β (with GAPDH as the internal control) in the Hela cells, which were treated with cisplatin and enzastaurin; (**E**, **F**) Ratio of p-PKC β to PKC β, Bcl-2 to GAPDH in the cisplatin- and enzastaurin-treated Hela cells. Data are mean ± S.E.M. of three independent experiments; ns, no significance; *P<0.05; **P<0.01 or ***P<0.001.

To discover the effect of cisplatin on PKC β and apoptosis related molecule: Bcl-2, Hela cells at 85% confluence were treated by 0, 10, 20 or 50 μM cisplatin, and 0 or 1 μM enzastaurin for 24 h. Then the expressions of PKC β, Bcl-2, p-PKC β, and internal control GAPDH in transfected cells were determined with WB. As shown in Figure 1A, single cisplatin treatment improved the p-PKC β level (Figure 1B) and decreased the Bcl-2 expression (Figure 1C). After quantifying the amount of each band, we calculated the ratio of p-PKC β/PKC β and Bcl-2/GAPDH respectively. We found that 10 μM, cisplatin treatment up-regulated the ratio of p-PKC β/ PKC β by two times, five times and four times with statistical differences (*P*<0.05, *P*<0.01 or *P*<0.001). With respect to the Bcl-2/GAPDH, treatment with 20 μM, 50 μM cisplatin down-regulated the index by 1.8-fold and 1.5-fold individually, accompanied with statistical differences (*P*<0.01 respectively). It seems that 20 μM cisplatin-treated group presented the peak ratio of p-PKC β/PKC β and the minimum Bcl-2/GAPGH value.

Subsequently, we tested the expressions of p-PKC β, PKC β and Bcl-2 at the different dose of enzastaurin and cisplatin. After WB analysis of each protein with the internal control GAPDH (Figure 1D), we found that single treatment of 20 μM of cisplatin induced the lowest Bcl-2 level and the highest p-PKC β level in treated Hela cells. The p-PKC β/PKC β value was not down-regulated by 1 μM enzastaurin treatment (Figure 1E). The ratio of p-PKC β/PKC β treated by 20 μM of cisplatin presented the top level among several groups with statistical differences (*P*<0.001, Figure 1E). As to the value of Bcl-2/GAPDH, 20 μM of cisplatin treatment decreased it by 50%, as was inhibited by 1 μM enzastaurin, with statistical differences (*P*<0.05, Figure 1F). The results also showed that 1 μM enzastaurin treatment had no effect on p-PKC β/ PKC β and Bcl-2/GAPDH levels.

### PKC β inhibitor down-regulates the cisplatin-induced apoptosis in treated Hela cells

**Figure 2:**
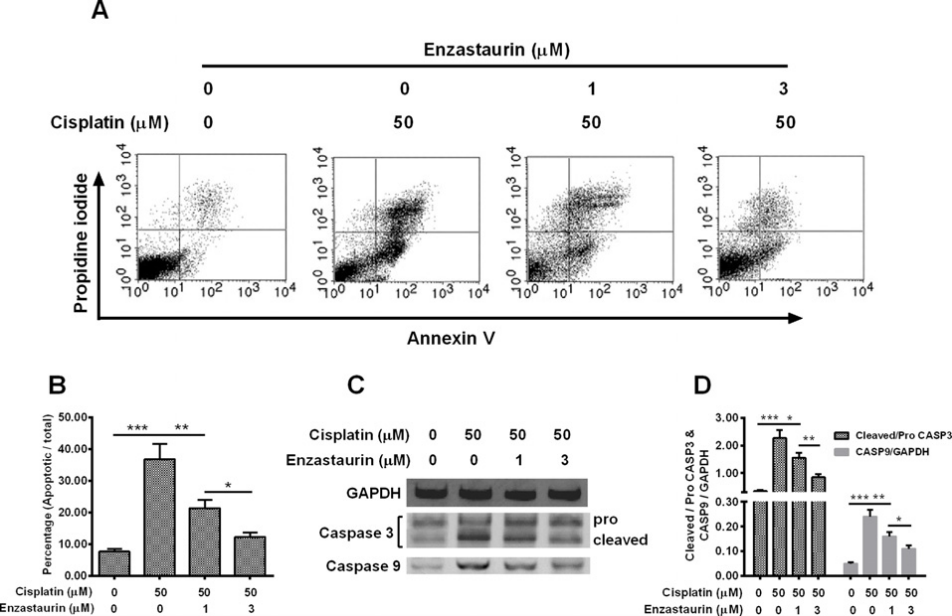
PKC β inhibitor down-regulates the cisplatin-induced apoptosis in Hela cells Hela cells were treated with 0 or 50 μM cisplatin, with 0, 1 or 3 μM PKC β inhibitor, enzastaurin for 24 h, then the apoptosis of Hela cells were assayed by flow cytometry with Annexin V-FITC Apoptosis Detection Kit (**A**) and was presented as a percentage of apoptotic cells to total cells (**B**), the apoptosis-related markers were examined by Western blotting method (**C**) and were presented as a relative levels to GAPDH (D). Experiments were performed independently in triplicate; *P<0.05, **P<0.01 or ***P<0.001.

To investigate the effect of PKC β inhibitor on the cisplatin-induced autophagy in cervical cancer cells, Hela cells were treated with 0 or 50 μM cisplatin in combination with 0, 1 or 3 μM enzastaurin for 24 h, then the apoptosis of Hela cells were assayed by flow cytometry with Annexin V-FITC Apoptosis Detection Kit. As indicated in Figure 2A,B, over 35% of cells treated by 50 μM cisplatin were induced with apoptosis. In comparison with cisplatin treatment alone, only 22% of cells were induced apoptosis with a statistical difference (*P*<0.01), which were treated by 50 μM cisplatin plus 1 μM enzastaurin. An enzastaurin dose-dependent decrease was observed. Treatment of 50 μM cisplatin plus 3 μM Enzastaurin presented <15% apoptotic rate, which displayed a statistical difference (*P*<0.05) when compared with the group treated by 50 μM cisplatin plus 1 μM enzastaurin.

We detected the expressions of apoptosis related molecules including caspase 3 (pro and cleaved) and caspase 9 from these groups (Figure 2C), GAPDH was set as the internal control. After quantifying the protein amount of each band, we found that enzastaurin treatment alone caused the highest ratio of caspase 9/GAPDH, while treatment of 50 μM cisplatin plus 3 μM enzastaurin display the lowest value of caspase 9/GAPDH among the treated groups with statistical differences (Figure 2D). As to the caspase3, cells treated with 50 μM cisplatin plus 3 μM enzastaurin show the lowest level of cleaved caspase3/pro-caspase3 among groups with statistical differences (Figure 2D), indicating that this dosage of enzastaurin could efficiently inhibit the cisplatin-induced apoptosis.

### PKC β inhibitor enhances the cisplatin-induced autophagy in Hela cells

**Figure 3:**
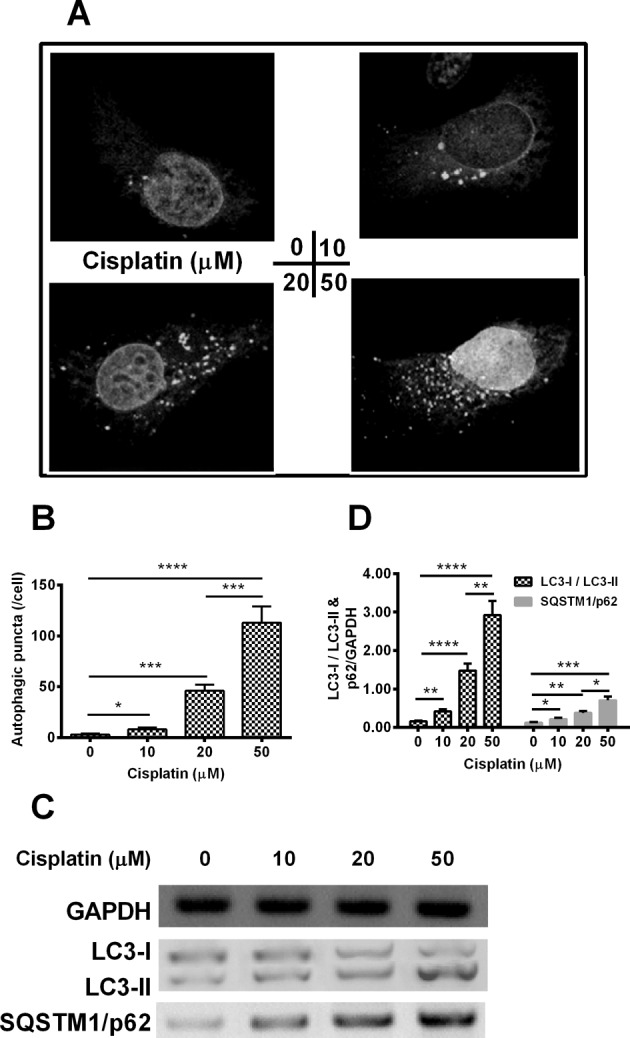
PKC β inhibitor enhances the cisplatin-induced autophagy in cervical cancer Hela cells Hela cells were transfected with EGFP–LC3 II reporter plasmid for 6 h and were treated with 0 or 20 μM cisplatin and with 0, 1 or 3 μM enzastaurin for 24 h, then Hela cells were observed under fluorescence microscopy. The image (**A**) and the number (**B**) of autophagic puncta were presented/counted respectively. And the autophagy-associated markers, such as microtubule-associated protein 1 light chain 3 (LC3)-I/II and autophagy protein 5 (ATG 5) were analysed by Western blotting assay (**C**) and were presented as ratio (**D**) of LC3-II/I and Atg 5 to GAPDH. Experiments were performed independently in triplicate; ns, no significance; * *P*<0.05, **P<0.01, *** *P*<0.001 or ****P<0.0001.

To discuss the function of PKC β inhibitor on the cisplatin-induced autophagy in cervical cancer Hela cells. Hela cells were transfected with EGFP–LC3 II reporter plasmid for 6 h and were treated with 0 or 20 μM cisplatin and with 0, 1 or 3 μM enzastaurin for 24 h, then were observed under fluorescence microscopy. As displayed in Figure 3A, obviously, the transfected cells treated by 200 μM cisplatin combined with 3 μM enzastaurin presented the highest number of puncta in one cell. The group cells thet were treated by 20 μM cisplatin plus 3 μM enzastaurin presented an average of 125 punctas per cell, which was the highest among groups (*P* <0.05, *P*<0.01 or *P*<0.0001, Figure 3B), an enzastaurin dose-dependent increase in autophagic puncta was observed as well.

We detected the expressions of LC3-I, LC3-II and Atg5 in the treated cells (Figure 3C) in the next step. With respect to the LC3-II/LC3-I, an enzastaurin-dependent increase was found among groups, which was approximately three when receiving 20 μM cisplatin plus 3 μM enzastaurin, cisplatin treatment alone only has a ratio of 0.5 and cells treated by 20 μM cisplatin plus 1 μM enzastaurin presented an average value of 1.5 (*P*<0.05, *P*<0.01, *P*<0.001 or *P*<0.0001, Figure 3D). The same trend was also observed in the Atg5/GAPDH, group dealt with 20 μM cisplatin plus 3 μM enzastaurin presented the highest value in the different groups (Figure 3D). All of the results indicated that PKC β inhibitor enhances the cisplatin-induced autophagy in cervical cancer Hela cells.

### Autophagy down-regulated the sensitivity of Hela cell to cisplatin

**Figure 4:**
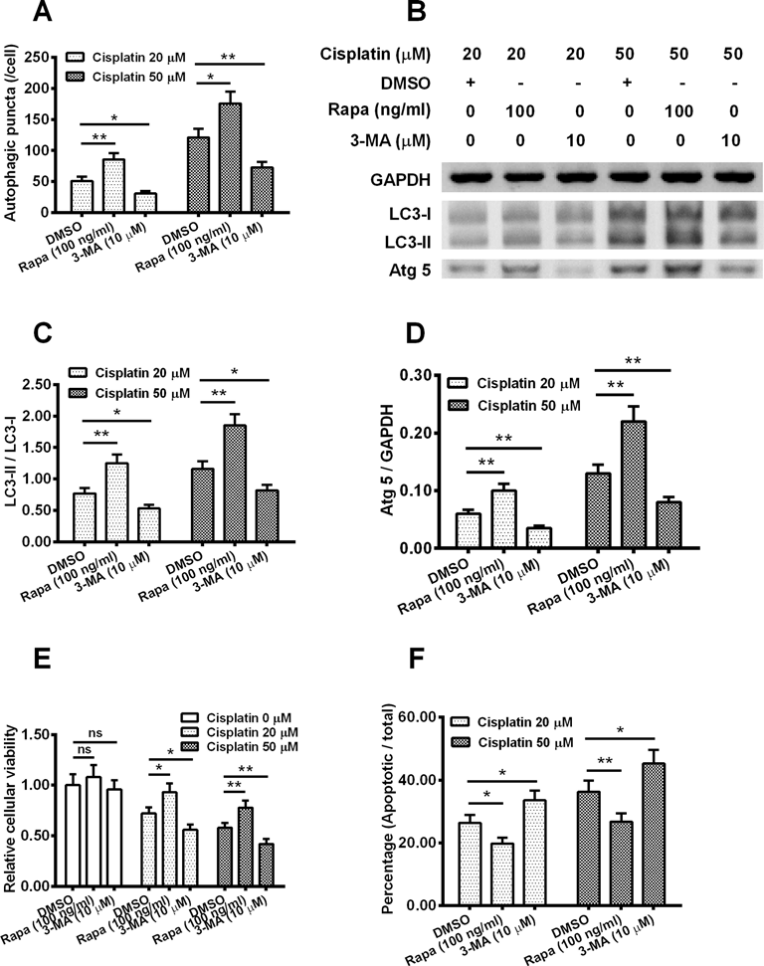
Influence by autophagy on the sensitivity of Hela cells to cisplatin (**A**) Autophagic puncta in the Hela cells, which were treated with 100 ng/ml Rapa or with 10 μM 3-MA and then with 20 or 50 μM cisplatin for 24 h; (**B**) Western blotting assay of LC3-I/II and Atg 5 in the Hela cells, post the Rapa (or 3-MA) treatment and the cisplatin treatment; (**C**, **D**) Ratio of LC3-II/I (C) and Atg 5 to GAPDH (D) in the Rapa (or 3-MA) and cisplatin treated Hela cells; (**E**) MTT assay for the cellular viability of the Hela cells, post the Rapa (or 3-MA) treatment and the cisplatin treatment; (**F**) Apoptosis induction of the Hela cells, post the treatment with Rapa (or 3-MA), in the presence of 0, 20 or 50 μM cisplatin. Experiments were performed independently in triplicate; ns, no significance; *P<0.05 or **P<0.01.

To evaluate the effect of autopagy on the Hela cell sensitivity to cisplatin, Hela cells were treated with 100 ng/ml Rapa or with 10 μM 3-MA and then with 20 or 50 μM cisplatin for 24 h, then autophagy puntics per cell were calculated. As shown in Figure 4A, statistical differences were found among the treated groups, whether with 20 μM cisplatin or with 50 μM cisplatin. In detail, Rapa (100 ng/ml) increased the autophagy punctas per cell by two-fold and 1.5-fold separately, when with 20 μM cisplatin or 50 μM cisplatin (*P*<0.05 or *P*<0.01).

We also measured the expressions of LC3-I, LC3-II and Atg5 using Western blotting (Figure 4B), we found cells treated by 100 ng/ml Rapa also presented the highest ratios of LC3-II/LC3-I, both at 20 μM cisplatin and 50 μM cisplatin. In detail, at the 20 μM cisplatin, Rapa treatment improved the average LC3-II/LC3-I value from 0.5 to 1.25 (*P* <0.01, Figure 4C) compared with the DMSO group and 3-MA treatment decreased the LC3-II/LC3-I ratio by 33% in comparison with the DMSO group (*P* <0.05, Figure 4C). As for the 20 μM cisplatin treatment, similar results were observed, Rapa efficiently up-regulated the LC3-II/LC3-I ratio and 3-MA down-regulated this parameter with statistical differences.

With respect to the Atg5/GAPDH and relative cellular viability, analogical results were acquired. Rapa improved these two parameters compared with the DMSO control, while 3-MA decreased them in contrast with DMSO group, both at 20 μM cisplatin or 50 μM cisplatin dosages, with statistical differences (*P*<0.05 or *P*<0.01, Figure 4D and Figure 4E). At last, we checked the apoptotic rate of different group, it was found that Rapa reduced the apoptotic rates by 32% (20 μM cisplatin, *P*<0.05) and 38% (50 μM cisplatin, *P*<0.01) respectively. In contrast, apoptotic rates were improved by 40% (*P*<0.05) and 20% (*P*<0.05) at 20 μM cisplatin and 50 μM cisplatin, when compared with the DMSO control, separately (Figure 4F).

### Overexpression of PKC β down-regulates the cisplatin-induced autophagy in Hela cells

**Figure 5:**
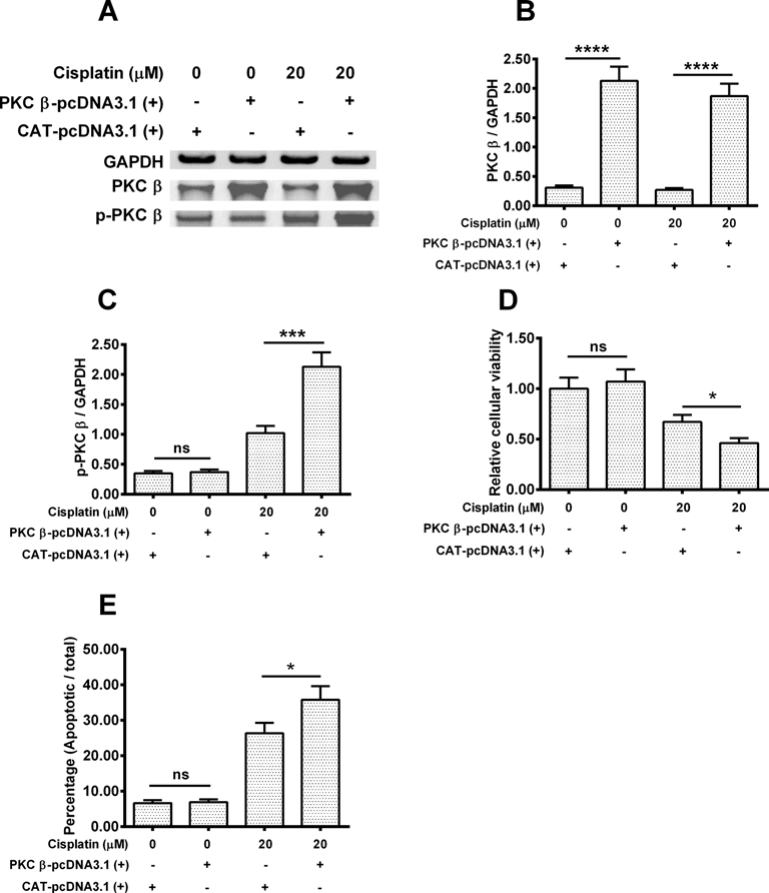
PKC β overexpression up-regulates the cisplatin-induced PKC beta phosphorylation and apoptosis in Hela cells Hela cells were transfected with PKC β-pcDNA3.1(+) or CAT-pcDNA3.1(+) plasmid for 6 h and then were treated with 0 or 20 μM cisplatin for 24 h. (**A**) Western blotting assay for PKC β, with or without phosphorylation (T642) in the Hela cells, with or without PKC β overexpression, in the absence of presence of cisplatin. (**B**, **C**) Ratio of PKC β (B) or p-PKC β to GAPDH in the Hela cells, with or without PKC β overexpression, in the absence of presence of cisplatin; (**D**, **E**) MTT assay for the cellular viability (D) or the apoptosis induction (E) of the Hela cells, post the PKC β overexpression and cisplatin treatment. Experiments were performed independently in triplicate; ns, no significance; *P<0.05, ***P<0.001 or ****P<0.0001.

To test whether the overexpression of PKC β would have an effect on the cisplatin-induced autophagy in Hela cells. Hela cells were transfected with PKC β-pcDNA3.1(+) or CAT-pcDNA3.1(+) plasmid for 6 h and then were treated with 0 or 20 μM cisplatin for 24 h. Then Western blotting assay for PKC β, p-PKC β and the internal control GAPGH was performed (Figure 5A). After quantifying the protein amount of each band, PKC β-pcDNA3.1(+) transfection indeed improve the PKC β expression (*P*<0.001) , even under the 20 μM cisplatin pressure(Figure 5B). Regarding the p-PKC β, PKC β-pcDNA3.1(+) transfection alone did not increase the level of p-PKC β/GAPDH (*P*>0.05), while up-regulated the p-PKC β/GAPDH level by nearly two times (*P*<0.001) when treated by 20 μM cisplatin (Figure 5C).

In the next step, we checked the cellular viability of each group, no statistical difference was observed between the PKC β-pcDNA3.1(+) transfected group alone and the CAT-pcDNA3.1(+) tranfected group alone, while cisplatin plus PKC β-pcDNA3.1(+) down-regulated the cellular viability by 33% in comparison with the group treated by cisplatin plus CAT-pcDNA3.1(+) (*P* <0.05, Figure 5D). In contrast, as indicated in Figure 5E, the apoptotic rate of the group treated by cisplatin combined with PKC β-pcDNA3.1(+) was improved by 40% compared with that of the cisplatin plus CAT-pcDNA3.1(+) treated group (*P*<0.05).

### Overexpression of PKC β reduces the cisplatin-induced autophagy in Hela cells

**Figure 6:**
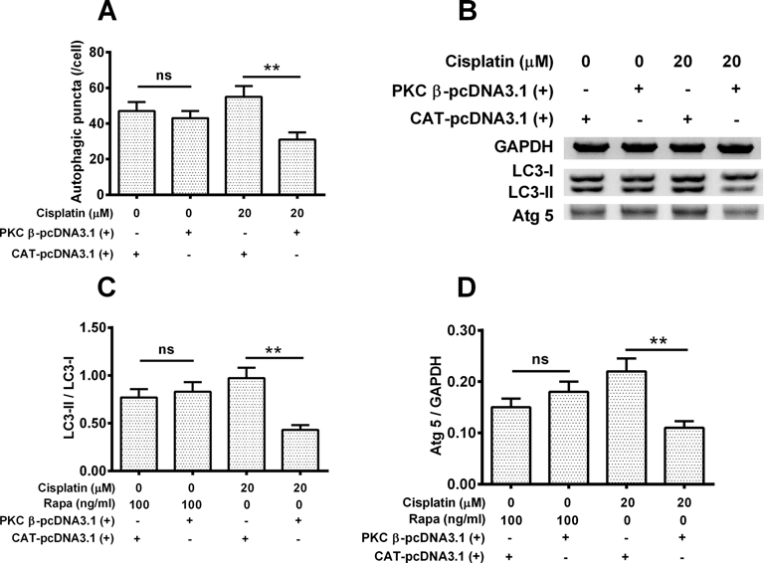
PKC β overexpression down-regulates the cisplatin-induced autophagy in Hela cells Hela cells were treated with 20 μM cisplatin or 100 ng/ml Rapa for 24 h, with or without PKC μ overexpression, then the autophgy level was assayed in each group of cells. (**A**) The number of autophagic puncta counted respectively; (**B**) Western blotting assay for LC3-I/II and ATG 5; (**C**, **D**) The ratio of LC3-II/I (C) and the ratio of Atg 5 to GAPDH (D). Data were presented as mean ± S.E.M. for triple independent experiments; ns, no significance; **P<0.01.

To explore whether overexpression of PKC β has an effect on the cisplatin-induced autophagy. Hela cells were treated with 20 μM cisplatin or 100 ng/ml Rapa for 24 h, with or without PKC β overexpression, then the autophgy level was assayed in each group of cells. As shown in Figure 6A, cells treated by cisplatin plus PKC β-pcDNA3.1(+) presented average 30 autophagic punctas per cell, which is lower than those treated by cisplatin plusCAT-pcDNA3.1(+), which had average 50 autophagic punctas per cell (*P*<0.05). We determined the expressions of LC3-I, LC3-II and Atg5 (Figure 6B) as well, GAPDH served as control. As indicated in Figure 6C, in the Rapa treated group, no difference was observed regarding the value of LSC3-II/LSC3-I between PKC β-pcDNA3.1(+) group and CAT-pcDNA3.1(+) group. Under the cisplatin treatment, the ratio of LCS-II/LSC3-I was down-regulated by 60% in PKC β-pcDNA3.1(+) group when compared with CAT-pcDNA3.1(+) group with a statistical difference (*P*<0.01). Similar results were also found in the parameter of Atg5/GAPDH (Figure 6C), no difference was observed between the group of Rapa plus PKC-β-pcDNA3.1(+)and the group of Rapa plus CAT-pcDNA3.1(+), and Atg5/GAPDH was greatly decreased in cisplatin combined with PKC β-pcDNA3.1(+) compared with that of cisplatin synergized with CAT-pcDNA3.1(+) (*P*<0.01, Figure 6D).

## Discussions

In the present study, we found that cisplatin promoted PKC β expression in Hela cells. Treatment of PKC β inhibitor reduced the cisplatin-induced apoptosis, whereas improved the cisplatin-induced autophagy. On the other hand, the PKC β overexpression aggravated the cisplatin-induced apoptosis, whereas down-regulated the cisplatin-induced autophagy. The exact role of autophagy in the process of tumour generation and progression is controversial either by promoting tumour cell survival or by inducing cell death [19,20]. 3-MA is a specific inhibitor of autophagy, it inhibits autophagy through inhibiting class-III PI3K in mammalian cells [21–23]. Here, we revealed the autophagy inhibitor 3-MA could improve the cells’ sensitivity to cisplatin (Figure 4E,F), while autophagy activator Rapa improved the sensitivity to cisplatin. We also found Rapa treatment decreased the apoptotic rate of cisplatin-treated cells, indicating that cisplatin inhibits the tumour cells by inducing apoptosis. It is supposed that autophagy inhibitors might sensitize tumour cells to cisplatin by improving the apoptotic rate induced by cisplatin or by changing the autophagic status to the apoptotic process.

Enzastaurin specifically disrupts the intrinsic phosphotransferase activity of PKC β at low concentrations, yet it inhibits other PKC isozymes at higher concentrations [21–23 ]. PKC β activates the MAPK and ERK cascade, affecting endothelial proliferation subsequently [24, 25]. Experiments on ovarian cell line indicated that enzastaurin is effective in suppressing taxane-resistant but not cisplatin-resistant ovarian tumours [26]. Enzastaurin induces apoptosis and suppresses proliferation in a range of cultured tumour cell lines through the PI3K–Akt pathway [27]. In the present study, we found that enzastaurin treatment presented a dose-dependent suppression of the apoptotic rate of cisplatin-treated cells. Above all, we also found that enzastaurin improved the autophagic puncta per cell in the cisplatin-treated cells with a dose-dependent increase. Key molecules Atg5 and LC3-II were also improved. These results indicate that enzastaurin efficiently induces the autophagy in treated cells and this should be noted in the clinical therapy.

Autophagy not only represents a survival mechanism to anticancer drugs but also can potentially lead to tumour cell death, which is also called type II programmed cell death (PCD) when autophagy exceeds a certain threshold [28,29]. It has been reported that Rapa exerts antitumour effect on malignant glioma cells by inducing autophagy, with a mechanism through inhibiting mTOR-signalling pathways [30,31]. Our data suggest that the PKC β inhibitor reduced the cisplatin-induced apoptosis, whereas increased the cisplatin-induced autophagy in Hela cells. In contrast, the PKC β overexpression aggravated the cisplatin-induced apoptosis, but down-regulated the cisplatin-induced autophagy. From these results, we can conclude that PKC β is ideal to sensitize cervical cancer cells to chemotherapy via reducing the chemotherapy-induced autophagy. Whether PKC β overexpression at a higher level could directly suppress Hela cells proliferation by inducing autophagy is to be explored.

In conclusion, our study firstly explored the involvement of PKC β in the cytotoxicity of cisplatin via inhibiting autophagy in cervical cancer cells. We propose that PKC β would sensitize cervical cancer cells to chemotherapy via decreasing the chemotherapy-induced autophagy in cancer cells. It will be a promising candidate for developing novel cisplatin-based chemotherapy against different kinds of tumours.

## Abbreviations

3-MA: 3-methyladenine
ATG 5: autophagy protein 5
Bcl-2: B-cell lymphoma 2
DMEM: Dulbecco’s modified Eagle’s medium
GAPDH: glyceraldehyde-3-phosphate dehydrogenase
LC3: light chain 3
PKC: protein kinase C
Rapa: rapamycin
mTOR: the mammalian target of rapamyin
MAPK: mitogen-activated protein kinase
ERK: extracellular regulated protein kinase
PI3K-Akt: phosphoinositide-3-kinase-RAC-alpha serine/threonine-protein kinase
ROS: reactive oxygen species
TNFalpha: tumor necrosis factor alpha
WB: western blotting

## References

[1] Dasari S. and Tchounwou P.B. (2014) Cisplatin in cancer therapy: molecular mechanisms of action. Eur. J. Pharmacol. 740, 364–378 25058905 10.1016/j.ejphar.2014.07.025 PMC4146684

[2] Loehrer P.J. and Einhorn L.H. (1984) Drugs five years later. Cisplatin. Ann. Intern. Med. 100, 704–713 6370067 10.7326/0003-4819-100-5-704

[3] Reedijk J. (2003) New clues for platinum antitumor chemistry: kinetically controlled metal binding to DNA. Proc. Natl. Acad. Sci. U.S.A. 100, 3611–3616 12655051 10.1073/pnas.0737293100 PMC152970

[4] Wozniak K. and Blasiak J. (2002) Recognition and repair of DNA-cisplatin adducts. Acta Biochim. Pol. 49, 583–596 12422229

[5] Wang H., Zhang G., Zhang H., Zhang F., Zhou B., Ning F. (2014) Acquisition of epithelial-mesenchymal transition phenotype and cancer stem cell-like properties in cisplatin-resistant lung cancer cells through AKT/β-catenin/Snail signaling pathway. Eur. J. Pharmacol. 723, 156–166 24333218 10.1016/j.ejphar.2013.12.004

[6] Basu A. and Krishnamurthy S. (2010) Cellular responses to cisplatin-induced DNA damage. J. Nucleic Acids 2010 10.4061/2010/201367 PMC2929606 20811617

[7] Ma B., Liang L.Z., Liao G.Q., Liang Y.J., Liu H.C., Zheng G.S. (2013) Inhibition of autophagy enhances cisplatin cytotoxicity in human adenoid cystic carcinoma cells of salivary glands. J. Oral Pathol. Med. 42, 774–780 23590333 10.1111/jop.12066

[8] Xu Y., Yu H., Qin H., Kang J., Yu C., Zhong J. (2012) Inhibition of autophagy enhances cisplatin cytotoxicity through endoplasmic reticulum stress in human cervical cancer cells. Cancer Lett. 314, 232–243 22019047 10.1016/j.canlet.2011.09.034

[9] Levine B. and Klionsky D.J. (2004) Development by self-digestion: molecular mechanisms and biological functions of autophagy. Dev. Cell 6, 463–477 15068787 10.1016/s1534-5807(04)00099-1

[10] Klionsky D.J. and Emr S.D. (2000) Autophagy as a regulated pathway of cellular degradation. Science 290, 1717–1721 11099404 10.1126/science.290.5497.1717 PMC2732363

[11] Katayama M., Kawaguchi T., Berger M.S. and Pieper R.O. (2007) DNA damaging agent-induced autophagy produces a cytoprotective adenosine triphosphate surge in malignant glioma cells. Cell Death Differ. 14, 548–558 16946731 10.1038/sj.cdd.4402030

[12] Carew J.S., Nawrocki S.T. and Cleveland J.L. (2007) Modulating autophagy for therapeutic benefit. Autophagy 3, 464–467 17495516 10.4161/auto.4311

[13] Blobe G.C., Obeid L.M. and Hannun Y.A. (1994) Regulation of protein kinase C and role in cancer biology. Cancer Metastasis Rev. 13, 411–431 7712599 10.1007/BF00666107

[14] Maioli E. and Fortino V. (2004) Protein kinase C: a target for anticancer drugs? Endocr. Relat. Cancer 11, 161–162 15163295 10.1677/erc.0.0110161

[15] Kouroedov A., Eto M., Joch H., Volpe M., Luscher T.F. and Cosentino F. (2004) Selective inhibition of protein kinase Cbeta2 prevents acute effects of high glucose on vascular cell adhesion molecule-1 expression in human endothelial cells. Circulation 110, 91–96 15210597 10.1161/01.CIR.0000133384.38551.A8

[16] Ishii H., Jirousek M.R., Koya D., Takagi C., Xia P., Clermont A. (1996) Amelioration of vascular dysfunctions in diabetic rats by an oral PKC beta inhibitor. Science 272, 728–731 8614835 10.1126/science.272.5262.728

[17] Kaushal G.P., Kaushal V., Herzog C. and Yang C. (2008) Autophagy delays apoptosis in renal tubular epithelial cells in cisplatin cytotoxicity. Autophagy 4, 710–712 18497570 10.4161/auto.6309

[18] Burman C. and Ktistakis N.T. (2010) Autophagosome formation in mammalian cells. Semin. Immunopathol. 32, 397–413 20740284 10.1007/s00281-010-0222-z

[19] Gatti L., Cossa G., Tinelli S., Carenini N., Arrighetti N., Pennati M. (2014) Improved apoptotic cell death in drug-resistant non-small-cell lung cancer cells by tumor necrosis factor-related apoptosis-inducing ligand-based treatment. J. Pharmacol. Exp. Ther. 348, 360–371 24345465 10.1124/jpet.113.210054

[20] Maiuri M.C., Tasdemir E., Criollo A., Morselli E., Vicencio J.M., Carnuccio R. (2009) Control of autophagy by oncogenes and tumor suppressor genes. Cell Death Differ. 16, 87–93 18806760 10.1038/cdd.2008.131

[21] Lum J.J., Bauer D.E., Kong M., Harris M.H., Li C., Lindsten T. (2005) Growth factor regulation of autophagy and cell survival in the absence of apoptosis. Cell 120, 237–248 15680329 10.1016/j.cell.2004.11.046

[22] Takasaka N., Araya J., Hara H., Ito S., Kobayashi K., Kurita Y. (2014) Autophagy induction by SIRT6 through attenuation of insulin-like growth factor signaling is involved in the regulation of human bronchial epithelial cell senescence. J. Immunol. 192, 958–968 24367027 10.4049/jimmunol.1302341

[23] Seglen P.O. and Gordon P.B. (1982) 3-Methyladenine: specific inhibitor of autophagic/lysosomal protein degradation in isolated rat hepatocytes. Proc. Natl. Acad. Sci. U.S.A. 79, 1889–1892 6952238 10.1073/pnas.79.6.1889 PMC346086

[24] Teicher B.A., Alvarez E., Menon K., Esterman M.A., Considine E., Shih C. (2002) Antiangiogenic effects of a protein kinase Cbeta-selective small molecule. Cancer Chemother. Pharmacol. 49, 69–77 11855754 10.1007/s00280-001-0386-2

[25] Mackay H.J. and Twelves C.J. (2007) Targeting the protein kinase C family: are we there yet? Nat. Rev. Cancer 7, 554–562 17585335 10.1038/nrc2168

[26] Cadron I., Van Gorp T., Mihalyi A., Luyten C., Drijkoningen K., Amant F. (2010) The impact of enzastaurin (LY317615.HCl) on CA125 biosynthesis and shedding in ovarian cancer cells. Gynecol. Oncol. 118, 64–68 20439112 10.1016/j.ygyno.2010.03.008

[27] Willey C.D., Xiao D., Tu T., Kim K.W., Moretti L., Niermann K.J. (2010) Enzastaurin (LY317615), a protein kinase C beta selective inhibitor, enhances antiangiogenic effect of radiation. Int. J. Radiat. Oncol. Biol. Phys. 77, 1518–1526 19906497 10.1016/j.ijrobp.2009.06.044 PMC3688843

[28] Chen N. and Karantza-Wadsworth V. (2009) Role and regulation of autophagy in cancer. Biochim. Biophys. Acta 1793, 1516–1523 19167434 10.1016/j.bbamcr.2008.12.013 PMC3155287

[29] Liu D., Yang Y., Liu Q. and Wang J. (2011) Inhibition of autophagy by 3-MA potentiates cisplatin-induced apoptosis in esophageal squamous cell carcinoma cells. Med. Oncol. 28, 105–111 20041317 10.1007/s12032-009-9397-3

[30] Iwamaru A., Kondo Y., Iwado E., Aoki H., Fujiwara K., Yokoyama T. (2007) Silencing mammalian target of rapamycin signaling by small interfering RNA enhances rapamycin-induced autophagy in malignant glioma cells. Oncogene 26, 1840–1851 17001313 10.1038/sj.onc.1209992

[31] Takeuchi H., Kondo Y., Fujiwara K., Kanzawa T., Aoki H., Mills G.B. (2005) Synergistic augmentation of rapamycin-induced autophagy in malignant glioma cells by phosphatidylinositol 3-kinase/protein kinase B inhibitors. Cancer Res. 65, 3336–3346 15833867 10.1158/0008-5472.CAN-04-3640

