# Expression of Concern: Protein kinase C β inhibits autophagy and sensitizes cervical cancer Hela cells to cisplatin

**DOI:** 10.1042/BSR20160445_EOC

**Published:** 2026-09-17

**Authors:** Na Li, Wei Zhang

**Affiliations:** Department of Gynaecology and Obstetrics, Zhongnan Hospital of Wuhan University, Wuhan 430071, China

**Keywords:** Autophagy, Chemotherapy, Cisplatin, Protein kinase C β

*Bioscience Reports* has been made aware of potential issues surrounding the scientific validity of this paper and is hence issuing a permanent Expression of Concern to notify readers. During an internal investigation, high similarity was identified between the Figure 6B GAPDH bands of this article and the Figure 5B GAPDH bands of Fang et al. 2016 (DOI: https://doi.org/10.1042/BSR20160118), as well as between the Figure 3C LC3-I 0 and 10 bands (lanes 1 and 2) within this article. The authors were contacted regarding the concerns but did not respond. As such, the article will remain published but with an associated Expression of Concern.

